# Metastatic breast tumors from extramammary malignancies: a case series

**DOI:** 10.1186/s40792-021-01235-2

**Published:** 2021-06-29

**Authors:** Ryoko Semba, Yoshiya Horimoto, Atsushi Arakawa, Mitsue Saito

**Affiliations:** 1 Department of Breast Oncology, Juntendo University School of Medicine, 2-1-1 Hongo, Bunkyo-ku, Tokyo, 113-0033 Japan; 2 Department of Human Pathology, Juntendo University School of Medicine, 2-1-1 Hongo, Bunkyo-ku, Tokyo, 113-0033 Japan

**Keywords:** Metastatic breast tumors, Extramammary malignancy, Ultrasound

## Abstract

**Background:**

Metastatic breast tumors from extramammary malignancies are quite rare. Characteristics of such tumors are unclear due to small number of reported cases. During 2012–2019, approximately 3,500 malignant breast tumors were diagnosed with needle biopsy at our hospital and we experienced three cases (0.09%) of metastatic extramammary malignancies. We herein report these cases focused on imaging and pathological findings.

**Case presentation:**

The first case was a 59-year-old woman who underwent curative surgery for thyroid cancer. After developing lung and ovarian metastases, she visited our department with a mass in her right breast. Ultrasound revealed a 7 mm-sized oval mass. With high depth–width ratio and abundant blood flow, primary breast cancer was suspected. Core needle biopsy revealed atypical cells with nuclear grooves proliferating in papillary formation. With immunohistochemical examination, her final diagnosis was metastatic thyroid cancer. The second case was a 74-year-old woman with metastatic spinal tumors and referred to our department for searching primary tumor. She was diagnosed with gastric cancer at the age of 41. Ultrasound revealed a hypoechoic area including cysts and the internal echo level was uneven. Contrast-enhanced magnetic resonance imaging showed a non-mass lesion with heterogeneous internal enhancement pattern, suggesting ductal carcinoma in situ. Core needle biopsy showed alveolar lesion with predominant signet cell-like morphology. We histologically diagnosed her disease as metastatic gastric cancer. The last case was 33-year woman with Stage IV clear cell sarcoma of the left foot. She came to our department after she felt a lump on her right breast. Ultrasound revealed a 45 mm-sized mass. Her disease was confirmed as metastatic clear cell sarcoma by needle biopsy.

**Conclusions:**

Imaging suggested malignancies, but it was difficult to distinguish them from primary breast cancer. Our cases indicate that metastatic tumors to the breast might have imaging patterns specific to primary organs, although more cases should be accumulated to establish such patterns on imaging. The first two cases shared some similar pathological findings with breast cancer, but also had some histological characteristics of the primary tumors. Hence, it was possible to diagnose these cases as metastatic tumors with careful observation.

## Background

Metastatic breast tumors from other malignancies are rare, 0.3–2.7% of all malignant breast tumors [1]. Most of these tumors are metastasized from the contralateral breast cancer, indicating cases metastasized from extramammary malignant tumors are even rarer. Metastasis to the breast might occur via both lymphatic and hematogenous [2, 3], but in any case, breast is the organ which is unlikely to metastasize. As primary tumors metastasizing to the breast, malignant melanoma, lung, ovarian, renal, and thyroid cancers are reportedly known [4].

Metastatic breast tumors merely form calcification and desmoplastic reaction on mammogram [5, 6]. However, characteristics of metastatic breast tumors are still unclear due to small number of previous reports.

During 2012–2019, approximately 3,500 malignant breast tumors were diagnosed with core needle biopsy at our hospital and we experienced three cases (0.09%) of metastatic tumor from extramammary malignancies. We herein report these three cases focused on imaging and pathological findings.

## Case presentation

### Case 1

**Fig. 1:**
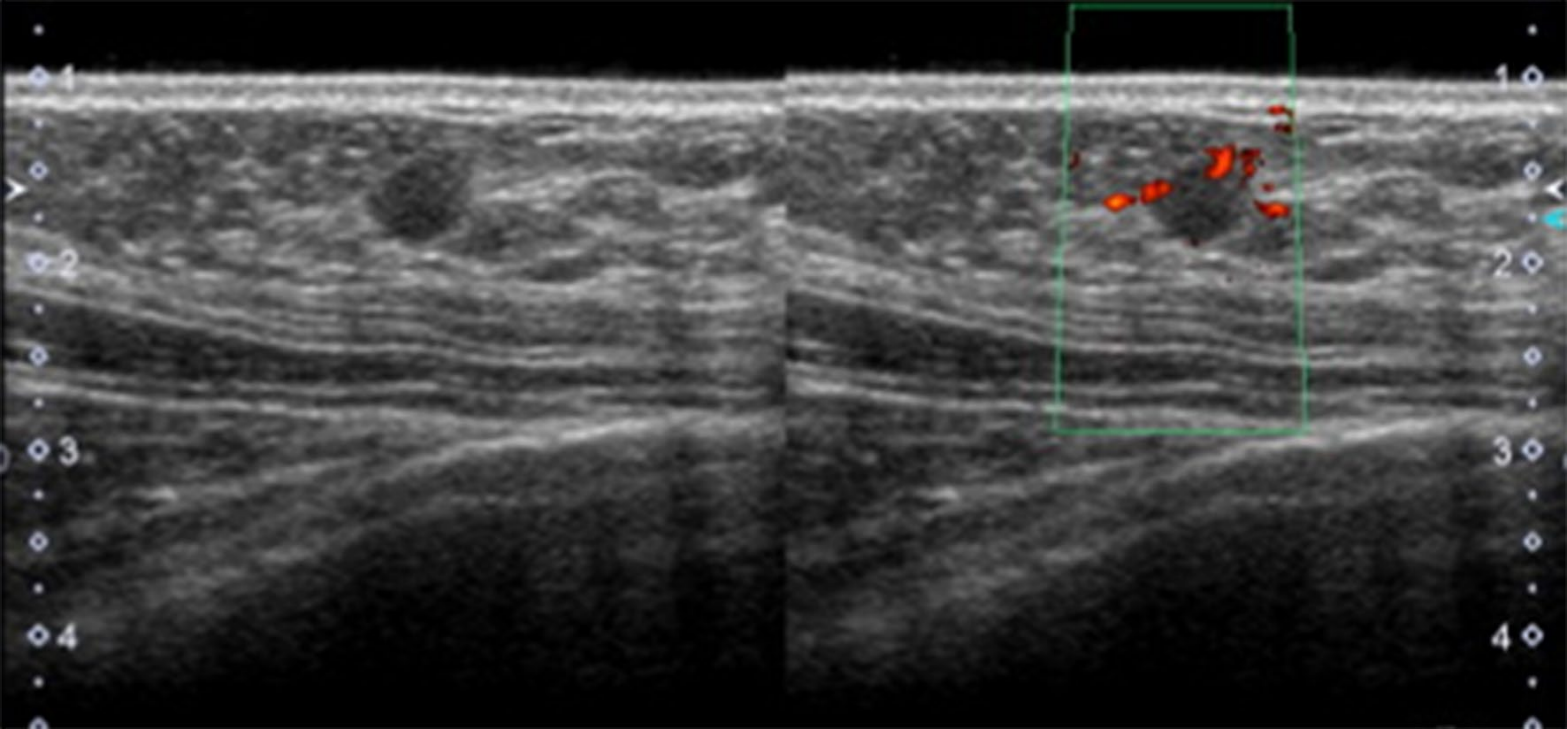
Ultrasound findings of case 1. Ultrasound showed a 7 mm-sized well-defined oval mass with a disrupted anterior border. Color Doppler revealed intra-tumoral abundant blood flow (right)

**Fig. 2:**
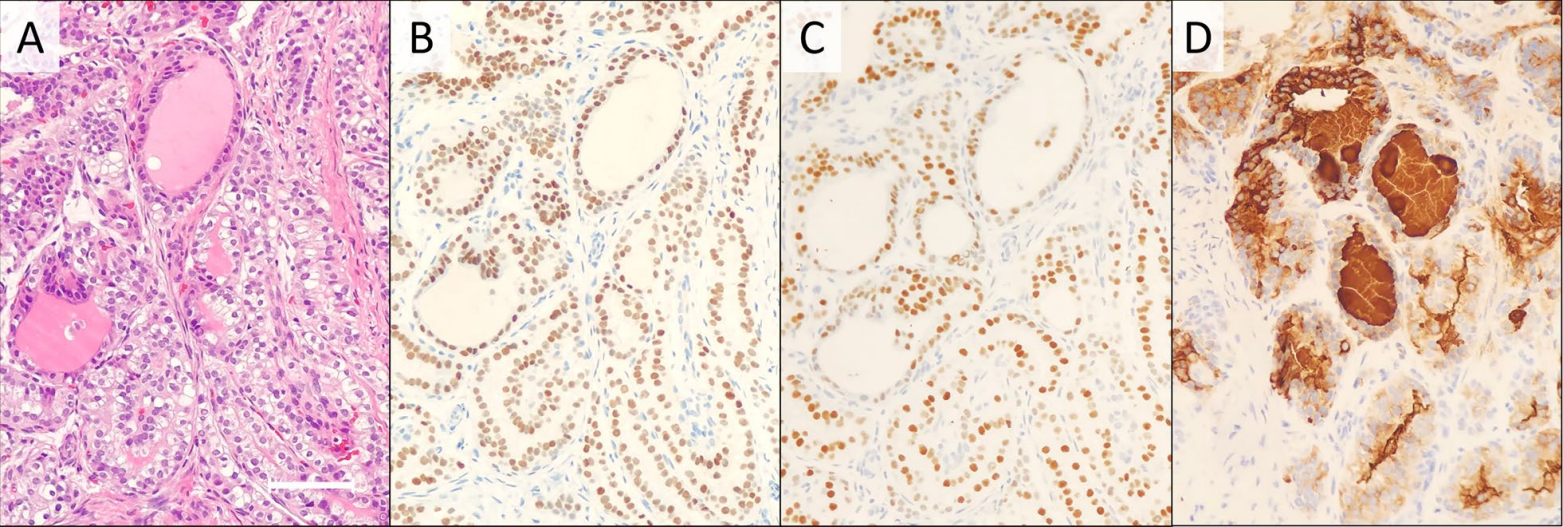
Pathological findings of needle biopsy. Core needle biopsy revealed papillary proliferation of atypical cells (**a**, hematoxylin and eosin staining ×200). Tumor cells were positive for both TTF-1 (**b**), Pax-8 (**c**) and thyroglobulin (**d**). A horizontal bar indicates 200 μm

A 49-year-old woman was diagnosed the left thyroid cancer (papillary carcinoma) and underwent subtotal thyroidectomy and lymph node dissection. One year later, she developed local recurrence in her right lobe and underwent completion thyroidectomy. Two years later, lung metastases were also found but it was resistant to internal radiation therapy with radioactive iodine. Skin metastases and ovarian metastases were also found after that. Ten years after initial surgery for thyroid cancer, she came to our department with her awareness of a mass in her right breast. Mammograms of both breasts showed no abnormal findings. Ultrasound revealed a 7 mm-sized oval mass located in the right upper region (Fig. 1). The internal echo level was mildly high and the posterior was iso- to hyperechoic. Depth width (D/W) ratio was high at 0.84, and blood flow was abundant. Based on these findings, her disease was suspected to be primary breast cancer, such as invasive breast carcinoma (no special type) and mucinous carcinoma. Other differential diagnoses included benign tumors, such as fibroadenoma and intraductal papilloma. Core needle biopsy was performed and histologically, atypical cells with nuclear grooves and intranuclear inclusions proliferated in papillary formation (Fig. 2). TTF-1, PAX-8 and thyroglobulin were all positive and the final diagnosis was metastatic carcinoma. The patient chose not to receive any therapy against cancer and have been receiving the best supportive care.

### Case 2

**Fig. 3:**
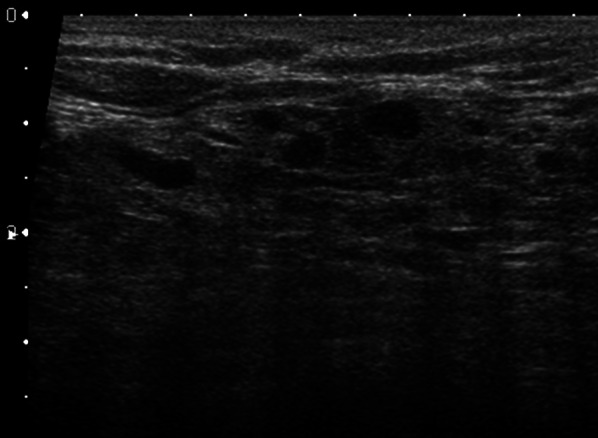
Ultrasound findings of case 2. Ultrasound showed a hypoechoic area with unclear boundaries including cysts and the internal echo level was uneven

**Fig. 4:**
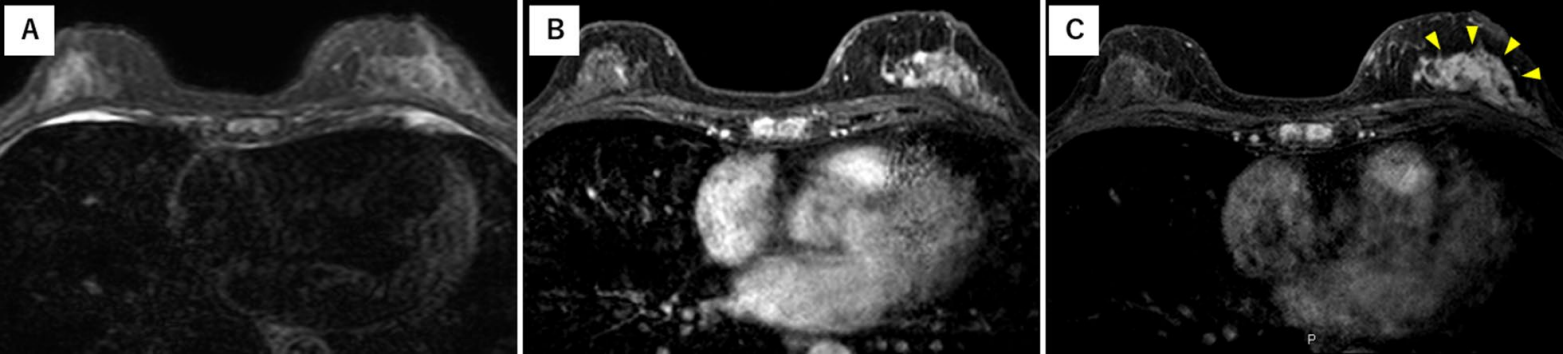
Findings of contrast-enhanced magnetic resonance. Contrast-enhanced MRI showed early enhancement of non-mass lesion in the upper-outer region of the left breast. **a** T2-weighted, **b** T1-turbo field echo (TFE) and **c** High-resolution (HR) images. TFE (**b**) and HR (**c**) images indicated abundant blood flow and early enhancement within the non-mass lesion (yellow arrowheads), respectively

**Fig. 5:**
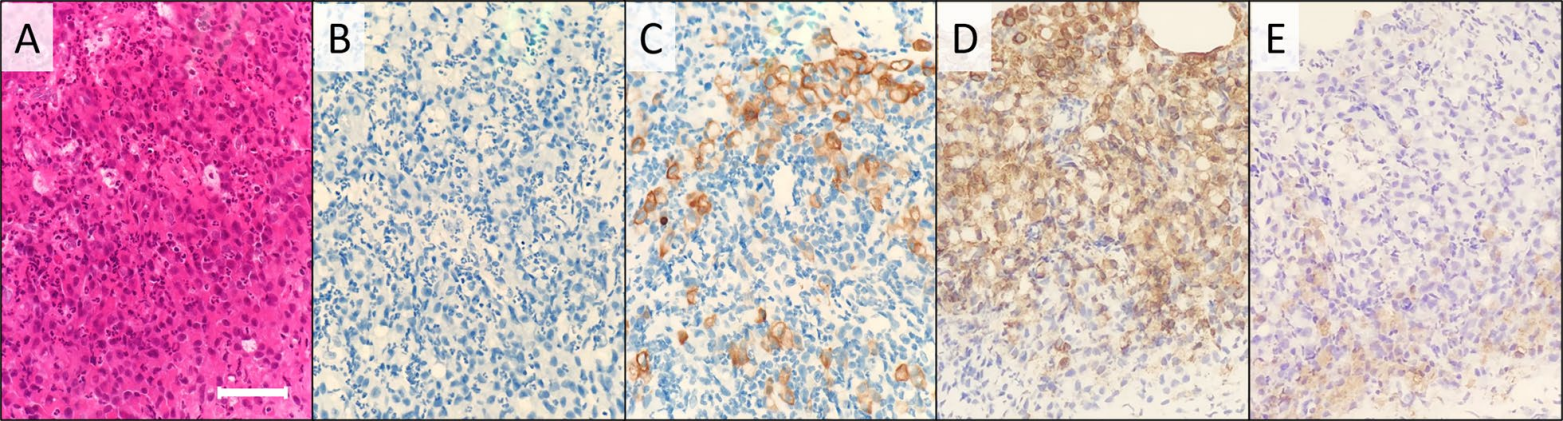
Pathological findings of needle biopsy. Incoherent atypical cells formed alveolar lesions with marked inflammation (**a**, hematoxylin and eosin staining, ×200). Tumor cells were negative for CK7 (**b**) and positive for CK20 (**c**), MUC5AC (**d**) and M-GGMC-1 (**e**). A horizontal bar indicates 100 μm

A 74-year-old woman was diagnosed with metastatic spinal tumors and received radiation therapy. She was diagnosed with gastric cancer at the age of 41 and her spinal tumors were suspected of gastric cancer metastases. She was referred to our department for searching primary tumor without any symptoms. Serum alkaline phosphatase was high but tumor markers were within normal limits. There were no specific findings on mammogram. Ultrasound revealed a hypoechoic area including cysts and the internal echo level was a mixture of low and high (Fig. 3). Contrast-enhanced magnetic resonance imaging (MRI) showed a non-mass lesion in the upper area of the left breast (Fig. 4). High-resolution (HR) imaging revealed internal enhancement pattern that was heterogeneous and clumped without obvious ductal spread. Based on these ultrasound and MRI findings, differential diagnoses were ductal carcinoma in situ (DCIS) of primary breast cancer and mastopathy. Core needle biopsy was performed and pathologic finding was a tumor forming alveolar lesion with highly active inflammation. Signet cell-like morphology was predominant and duct formation was partially observed. On immunohistochemistry, mammaglobin, GCDFP-15 and CK7 were all negative and CK20 was focally positive (ER < 5%, PgR < 5%, HER2-negative, Ki67 labelling index: 50%; Fig. 5). Tumor cells were positive for MUC5AC, while negative for MUC1, MUC2 and MUC6. This staining pattern corresponds to tumors arising from gastric glands. Moreover, M-GGMC-1 (anti-mucin monoclonal antibody recognizing gastric gland mucous cells) was also partially positive. Based on these results, we diagnosed metastatic adenocarcinoma from gastric cancer. She died after developing the disseminated intravascular coagulation (DIC) due to progression of gastric cancer.

### Case 3

**Fig. 6:**
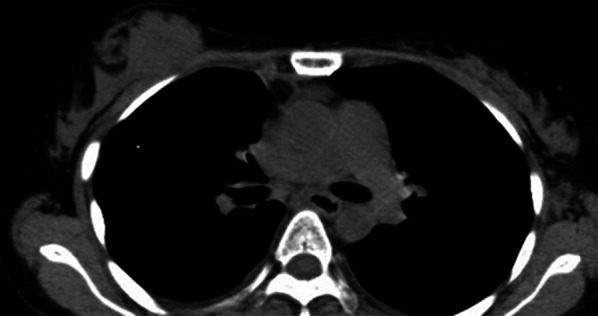
Findings of computed tomography of case 3. A 45 mm-sized lesion with an equal density to soft tissue was observed in the upper area of the right breast

**Fig. 7:**
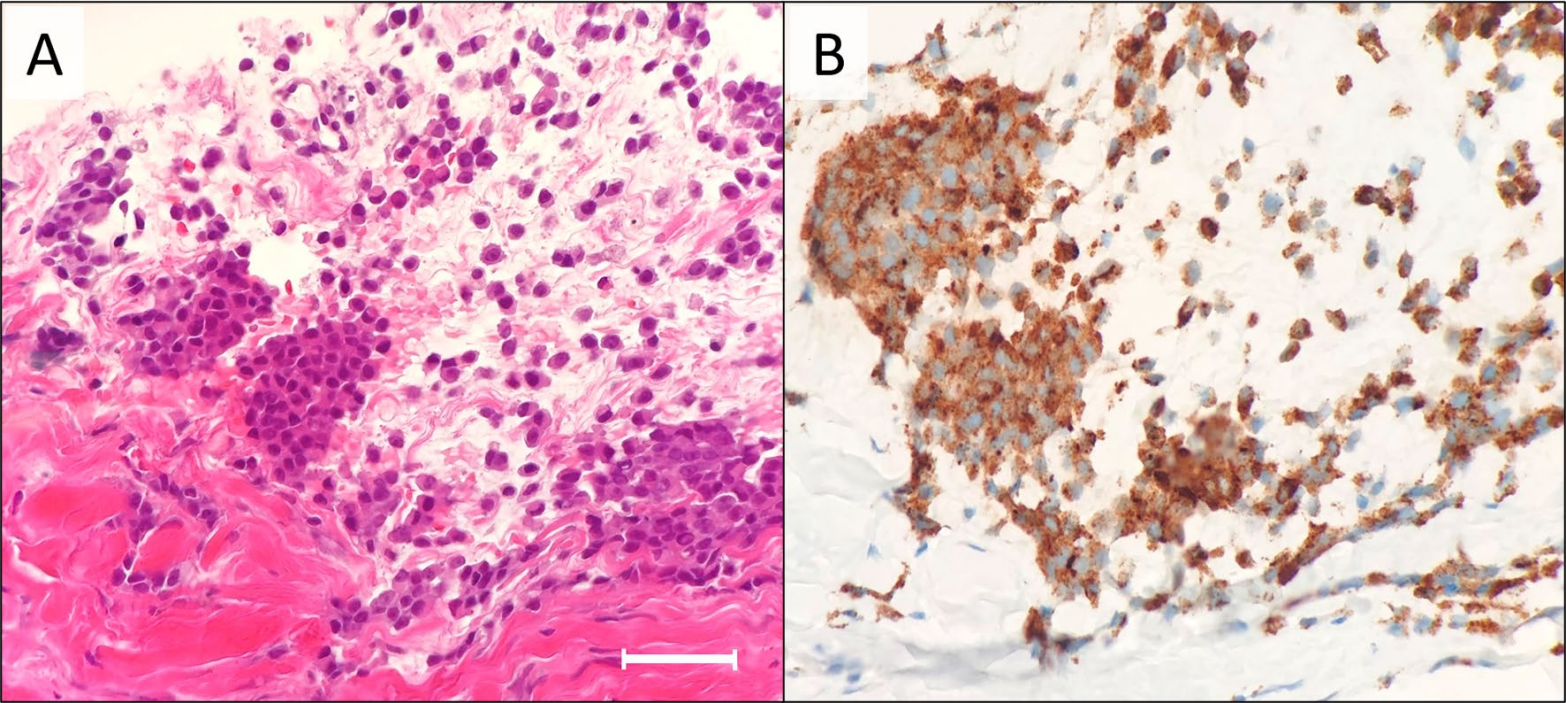
Pathological findings of needle biopsy. Atypical cells with clear cytoplasm were observed (**a**, hematoxylin and eosin staining, ×200). Lack of cell–cell adhesion is one of the key findings that indicate non-epithelial neoplasm. Tumor cells were positive for HMB45 (**b**). A horizontal bar indicates 100 μm

A 33-year-old woman was diagnosed with clear cell sarcoma of the left foot and underwent radical surgery for tumor resection. One year later, she developed lung metastases and underwent partial resection following chemotherapies. Then her clear cell sarcoma metastasized to upper mediastinal lymph nodes, pulmonary pleura, left thigh, and back lumbar area. One year and 8 months after the initial diagnosis of clear cell sarcoma, she came to our department, because she felt a lump on her right breast A 30 mm-sized mass was palpable in her right upper breast. Mammograms showed no significant findings. Ultrasound revealed a 45 × 45 × 21 mm-sized mass in the right breast (imaging data were not available). Computed tomography (CT) showed a soft tissue lesion in the upper part of her right breast (Fig. 6). It was difficult to judge whether the tumor was benign or malignant based on CT findings. Core needle biopsy was performed and she was diagnosed metastatic clear cell sarcoma, with both HMB45 and CD56 being positive and all negative for synaptophysin, chromogranin A, S-100, mammaglobin and GCDFP-15 (Fig. 7). She subsequently died 2 months later due to progression of her disease.

## Conclusions

None of our three cases had significant findings on mammogram, consistent with previous reports. Ultrasound and MRI findings suggested malignancies, but it was difficult to distinguish these tumors from primary breast cancer. Case 1 (thyroid cancer) and 2 (gastric cancer) were visualized as a well-defined lobular mass and a non-uniform hypoechoic region, respectively. Mun et al*.* indicated that metastatic breast tumors might have different imaging features depending on how primary malignant tumors metastasize, hematogenously or lymphatically [7]. According to the report, hematogenously metastasized tumors, typically head and neck cancers such as thyroid cancer [3], often form well-defined masses under the skin or near the parenchyma of the mammary gland, where blood flow is abundant. On the other hand, lymphatically metastasized tumors, typically gastrointestinal and gynecological cancers [3], appear with unclear boundaries. Our first two cases were consistent with these reports, in terms of image findings, although more cases should be accumulated to draw conclusions.

As to pathological differential diagnosis, papillary proliferation as seen in case 1 and duct formation as in case 2 can also commonly be observed in breast cancer, while case 3 had sarcomatous findings, apparently different from adenocarcinoma. However, both two cases of adenocarcinoma (case 1 and 2) also had histological characteristics of the primary tumors (e.g., nuclear grooves and intranuclear inclusions for thyroid cancer). Therefore, histologically, it was possible to diagnose these cases as metastatic tumors with careful observation. Nevertheless, it was crucial to obtain clinical information including disease history of other cancers, to perform proper and efficient immunohistochemistry.

## Data Availability

Not applicable.

## Abbreviations

CK: Cytokeratin
CT: Computed tomography
DIC: Disseminated intravascular coagulation
ER: Estrogen receptor
GCDFP-15: Gross cystic disease fluid protein-15
HER2: Human epidermal growth factor receptor 2
HMB45: Human melanin black 45
HR: High-resolution
MRI: Magnetic resonance image
PgR: Progesterone receptor
TFE: Turbo field echo
